# New Poly(lactide-urethane-isocyanurate) Foams Based on Bio-Polylactide Waste

**DOI:** 10.3390/polym11030481

**Published:** 2019-03-12

**Authors:** Joanna Paciorek-Sadowska, Marcin Borowicz, Marek Isbrandt

**Affiliations:** Department of Chemistry and Technology of Polyurethanes, Technical Institute, Faculty of Mathematics, Physics and Technical Science, Kazimierz Wielki University, J. K. Chodkiewicza Street 30, 85-064 Bydgoszcz, Poland; insttech@ukw.edu.pl

**Keywords:** PLA, rigid polyurethane-polyisocyanurate foams, recycling, mechanical properties, 3D printing waste

## Abstract

The article presents the results of research on the synthesis of a new eco-polyol based on polylactide (PLA) waste and its use for the production of rigid polyurethane-polyisocyanurate (RPU/PIR) foams. The obtained recycling-based polyol was subjected to analytical, physicochemical and spectroscopic tests (FTIR, ^1^H NMR, ^13^C NMR) to confirm its suitability for the synthesis of polyurethane materials. Then, it was used to partially replace petrochemical polyol in polyurethane formulation. The obtained RPU/PIR foams were characterized by lower apparent density, brittleness, and water absorption. In addition, foams modified by eco-polyol had higher flame retardancy, as compared to reference foam. The results of the research show that the use of PLA polyol based on plastic waste may be an alternative to petrochemical polyols. This research matches with the current trends of sustainable development and green chemistry.

## 1. Introduction

Rapid development of the polymer industry has been observed in recent years. In 2017, the global production of plastics amounted to 335 million tons, a 4% increase compared to the previous year. Such an upward trend shows that, in the near future, the global economy will face an increasing amount of polymer waste [1]. The most common ways of plastic waste management include energy, material, and chemical recycling. The popularity of these methods is increasing, and the processing of polymeric materials is being improved. At the same time, new legal regulations on environmental protection are forcing scientists and entrepreneurs to look for new solutions to manage plastic waste. Such requirements are pushing science and industry towards biodegradable plastics that decompose in the natural environment [2,3,4].

Biodegradable plastics have properties comparable to polymers of petrochemical origin. At the same time, they are completely biodegradable in the natural environment. An additional advantage of these materials is the fact that they are obtained from renewable sources, for example, from vegetable origin sources, such as corn, rice and biomass. Known technologies have made it possible to obtain between 200–300 grams of biopolymers per square meter of cultivated field. Currently, several types of biodegradable plastics are produced worldwide. The most developed research among them concerns polylactide (PLA) [5,6,7,8,9,10].

In terms of chemical structure, polylactide belongs to the group of aliphatic biopoly(esters), which are products of the self-esterification reaction of β-hydroxy acids. Due to properties similar to other synthetic polymers (such as polystyrene or polyethylene terephthalate) and the ability to be completely biodegradable, it is an interesting alternative to materials produced from refining products of crude oil [11,12,13].

The global production of polylactide has increased and now stands at 0.2 million tones, which is 10.3% of the total production of biodegradable polymers [14]. The largest producers are in Europe—BASF AG, Apack AG, Boeringer Ingelheim (Germany), Purac Biochem (The Netherlands), Phusis (France); North America—Dow Cargill, NatureWorks, Birmingham Polymers (USA); and Asia—Bio Invigor (Thailand) and Mitsui Chemicals (Japan) [6,15,16].

PLA can be processed by using standard equipment and technologies such as extrusion, thermoforming, injection molding, and extrusion blow molding [5]. This perspective entails many uses of polylactide—production of packaging, bottles, films, disposable products, orthopedic and dental implants, protective materials in civil engineering or agriculture and as a filament in 3D printing technology [17,18,19,20,21,22]. The high usage of this material results in very large amounts of biodegradable waste. The recovery of polymer raw materials from worn-out products is an important activity, which should be carried out on products whose useful life has ended [23]. The biodegradation process has positive and negative aspects. A big advantage is the fact that biodegradable polymers under composting conditions are completely decomposed into water, carbon dioxide or methane, and biomass. However, a disadvantage is the high cost of storage and the need to build a composting plant for biodegradable materials. In addition, as a result of the biodegradation process, the benefits of physical (no recyclability of the polymer and its secondary processing), energy (not using the calorific value of biopolymers) or chemical recycling (no recovery of monomers and oligomers and their reuse in the polymerization process) are not obtained. In summary, the composting process excludes the possibility of multiple use of biopolymers [5,20].

According to statistical data, in 2016, raw material recycling in Europe exceeded waste storage for the first time [1]. Therefore, alternative solutions are being sought for the processing of polylactide waste (mainly from 3D printing technology) to full-value products. There are solutions for the management of other forms of waste polyester (polyethylene terephthalate—PET), which is used for the synthesis of polyol raw materials for polyurethane systems. PET-based polyol was one of the cheapest polyol raw materials in the beginning of the 21st century. The increase in its consumption and emission of post-consumer waste caused a large increase in the interest in processing products of this polymer. Currently, polyols based on PET are an important branch of production of raw materials for polyurethanes. However, their price has increased four times in the last two decades [24,25,26]. Unfortunately, there is not much research on the use of PLA waste for synthesis of polyols for polyurethane materials.

The current challenge for the polyurethane industry is to eliminate petrochemical polyol raw materials for plant-derived biopolyols or recycled eco-polyols [27,28,29]. Products with a relatively low molecular weight are important for the synthesis of polylactide-based polyols. The presence of one hydroxyl group and one carboxyl group on the opposite ends of the molecule prevents direct use as a polyol raw material. Hence, the –COOH group should be functionalized in such a way as to obtain a hydroxyl derivative. Seppala et al. submitted a method involving the equimolar reaction of ethylene glycol with a carboxylic group of PLA oligomerol [30]. As a result of the synthesis, a liquid diol derivative of the polylactide with very low molecular weight (about 1700) was obtained. The obtained product met the basic polyol criterion, i.e., the presence of at least two hydroxyl groups. Wang et al. proposed the synthesis of poly(lactide)diol based on the reaction of lactide with 1,4-butanediol against the organotin catalyst. The obtained polyol was a low molecular weight solid (*M*_n_ = 3000–10,000), which was well soluble in hot toluene and 1,4-butanediol [31].

By combining the aforementioned aspects of PLA waste processing into a full-value product and the trend of addition of bio- or eco-polyols into polyurethane materials, the authors proposed a method of chemical recycling of polylactide waste from 3D printing technology to oligomeric polyhydric alcohols, investigating their impact on new polyurethane materials in the form of rigid polyurethane-polyisocyanurate (RPU/PIR) foams.

## 2. Materials and Methods

### 2.1. Raw Materials

**Figure 1:**
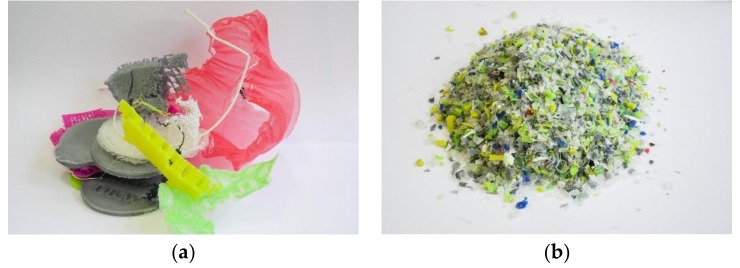
Appearance of polylactide waste: (**a**) after 3D printing, (**b**) after grinding.

A mixture of different ground bio-polylactide waste (Figure 1) from 3D printing and 98% diethylene glycol (Chempur, Piekary Śląskie, Poland) was used for synthesis of new polyol in accordance with Polish Patent Application P.424629. The glycolysis reaction catalyst was anhydrous zinc stearate (Chempur, Piekary Śląskie, Poland).

PLA-polyol based on waste products of 3D printing and polyol Rokopol RF-551—sorbitol oxyalkylation product with hydroxyl number HN = 420 mg KOH/g (PCC Rokita S.A., Brzeg Dolny, Poland) was used for synthesis of RPU/PIR foams. Purocyn B, a technical polyisocyanate (supplied by Purinova, Bydgoszcz, Poland) was used as the isocyanate raw material. Its main component was 4,4′-diphenylmethane diisocyanate. The content of the NCO group was equal to 31%.

The catalytic system for synthesis of RPU/PIR foams was made from anhydrous potassium acetate (Chempur, Piekary Śląskie, Poland), used in 33% solution in diethylene glycol (Chempur, Piekary Śląskie, Poland) and DABCO—1,4-diazabicyclo[2,2,2]octane (Alfa Aesar, Haverhill, MA, USA) used also in 33% solution in diethylene glycol. Tegostab 8460—polysiloxanepolyoxyalkylene surfactant (Evonik, Essen, Germany) was used as foam structure stabilizer. Carbon dioxide was used as a blowing agent. It was produced in situ by a reaction between distilled water and excess isocyanate raw material. Antiblaze TCMP—trichloro-2-methylethyl phosphate (Albemarle, Charlotte, NC, USA) was used as a flame retardant.

### 2.2. Glycolysis of 3D Printer PLA

**Figure 2:**
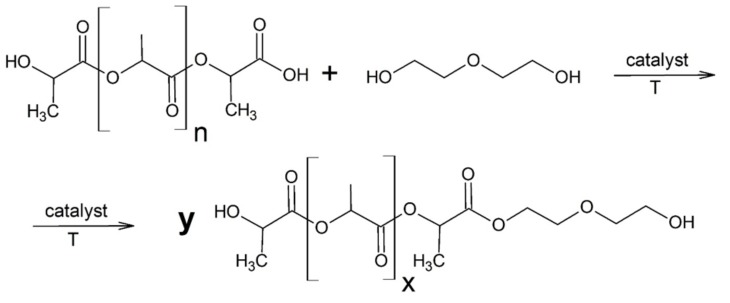
Glycolysis reaction of polylactide waste.

Ground PLA waste, 98% diethylene glycol and zinc stearate (mass of the reactants were shown in Table 1) were loaded into a reactor with a reflux condenser, thermometer, dropping funnel and mechanical stirrer. Mass ratio of PLA to pure glycol was 1:0.5. The mixture was heated to 200 °C with continuous mixing of the stirrer (700 rpm). Polylactide waste was liquefied at this temperature. The glycolysis reaction (Figure 2) was carried out for three hours, then the system was cooled. After cooling, the PLA-polyol was filtered and prepared for testing.

### 2.3. Examining the Properties of PLA-Polyol

#### 2.3.1. Analytical Tests

Physicochemical, analytical, and spectroscopic tests were performed on the new bio-polyol. This was to determine its suitability for the synthesis of RPU/PIR foams. 

The hydroxyl number (HN) was determined in accordance with Purinova Ltd. standards—WT/06/07/PURINOVA, by acylation method with acetic anhydride in *N*,*N*′-dimethylformamide, as a medium. An excess of acetic anhydride after hydrolysis and the obtained acetic acid were titrated by using a standard potassium hydroxide solution and phenolphthalein, as an indicator.

Acid value (AV) was determined in accordance with WT/06/07/PURINOVA. The analysis was performed by titration of the sample dissolved in acetone by using the standard solution of potassium hydroxide in ethyl alcohol and phenolphthalein as an indicator.

The viscosity of the PLA-polyol was determined by using a digital rheometer (Fungilab Inc., New York, NY, USA) at 20 °C (293 K). The measurements were carried out by using a standard spindle (DIN-87) working with the bushing (ULA-DIN-87). A thermostat connected to the water jacket of the sleeve helped maintain constant measurement temperature. 

Density was measured at 25 °C (298 K) in an adiabatic pycnometer in accordance with ISO 758:1976. 

The pH value was measured using microprocessor laboratory pH-meter (ORP/ISO/°C) with RS22C connector (Hanna Instruments, Woonsocket, RI, USA). 

Water content was determined by the Carl-Fisher method using a non-pyridine reagent with trade name Titraqual, in accordance with PN-81/C-04959.

Elemental analysis was done using the Vario EL III CHNSO analyzer (Elementar, Langenselbold, Germany).

The average molecular weight (*M*_n_) of the PLA-polyol was determined by Gel Permeation Chromatography (GPC) using a Knauer chromatograph (Knauer GmbH, Berlin, Germany). The apparatus was equipped with thermostated columns and a refractometer detector. The measurements were made on the basis of calibration, by using polystyrene standards in the range of *M*_n_ from 162 g/mol to 25,500 g/mol. On the basis of HN and average *M*_n_ of PLA-polyol, functionality (f) was calculated.

#### 2.3.2. Spectroscopy Tests

The PLA-polyol was tested using the Fourier transform infrared (FTIR) spectroscopy method and the Nicolet iS10 spectrophotometer (Thermo Fisher Scientific, Waltham, MA, USA) in the 400–4000 cm^−1^ range, and nuclear magnetic resonance spectroscopy ^1^H NMR and ^13^C NMR using a NMR Ascend III spectrometer (Brücker, Billerica, MA, USA) with a frequency of 400 MHz, in deuterated chloroform as a solvent.

#### 2.3.3. Differential Scanning Calorimetry (DSC)

Analysis of the differential scanning calorimetry (DSC) of PLA-polyol was carried out using DSC 204 F1 apparatus (Netzsch Analysing & Testing, Selb, Germany) in an atmosphere of inert nitrogen gas. The temperature range of measurement was from 20 °C to 200 °C and the heating rate was 10 °C/min. A sample with mass of 10 mg was used for the test. The polyol sample was not additionally dried before testing.

### 2.4. Preparation of RPU/PIR Foams

The preparation of RPU/PIR foams with PLA-polyol based on 3D printing waste required experimental investigations to determine the optimal composition of additive agents (surfactant, catalytic system, flame retardant and blowing agent). The hydroxyl number was the basis to determine the amount of polyol raw material. These values enabled calculation of the mass equivalent (*Eq*_OH_) of the commercial polyol and PLA-polyol. The addition of isocyanate raw material was selected taking into account the mass equivalent ratio of NCO to OH groups (*Eq*_NCO_:*Eq*_OH_) in the reaction mixture. For RPU/PIR foams blown by water, this ratio was 3.7:1. An excess of isocyanate raw material was necessary for a reaction between the NCO and OH groups to produce a urethane bond; trimerization reaction of three NCO groups to produce an isocyanurate ring; and reaction between NCO group and distilled water to produce in situ a blowing agent—carbon dioxide. The sum of mass equivalents of commercial polyol and PLA-polyol was always 1.

The content of additive agents was calculated in relation to the sum of masses of polyols and polyisocyanate (in weight percentages): urethane bond catalyst (1 wt %), isocyanate trimerization catalyst (2.5 wt %), chemical blowing agent (1 wt %), flame retardant (17 wt %) and structure stabilizer (1.7 wt %). The formulation of RPU/PIR foams modified by PLA-polyol is shown in Table 2.

The foams were made in a laboratory using a one-step method from two components—A and B. Component A was obtained as a result of mixing of appropriate amounts of polyols, catalysts, surfactant, blowing agent and flame retardant (according to Table 2). Component B was Purocyn B. Components A and B were mixed for 10 s by a mechanical stirrer (1800 rpm) in an appropriate mass ratio. The mixture was poured into a cuboidal mold with internal dimensions of 25 cm × 25 cm × 30 cm, producing foam. The synthesis of RPU/PIR foams was repeated twice. The obtained polyurethane materials were thermostated for six hours at 120 °C in a laboratory dryer with forced circulation, after removal from the mold.

### 2.5. Assessing the Properties of RPU/PIR Foams

#### 2.5.1. Processing Times

During the process, the characteristic foaming time was analyzed using an electronic stopwatch in accordance with ASTM D7487 13e1—Standard Practice for Polyurethane Raw Materials: Polyurethane Foam Cup Test [32]. During synthesis of the RPU/PIR foams, cream, free rise, string gel, and tack free times were measured.

#### 2.5.2. Selected Properties of New Composites

New PLA-based RPU/PIR composites were tested for their basic functional properties i.e., apparent density, brittleness, compressive strength, aging resistance, water absorption, and thermal insulation.

The apparent density of new foams was determined as the ratio of foam weight to its geometrical volume in accordance with ISO 845:2006.

Brittleness was determined in accordance with ASTM C-421-61, as a percentage weight loss of 12 cubic foam samples with a side length of 25 mm. Tests were conducted in a standard cuboidal box made of oak wood with dimensions of 190 mm × 197 mm × 197 mm, rotating around the axis at a speed of 60 rpm. During the measurement, the box was filled with 24 normalized oak cubes with dimensions of 20 mm × 20 mm × 20 mm.

Compressive strength was determined as maximum force inducing a 10% relative strain (according to the foam growth direction) on the known surface of the foam by using the universal testing machine Instron 5544 (Instron, Norwood, MA, USA) in accordance with ISO 844:2014.

Accelerated aging tests of the samples were carried out by a thermostating process for 48 h at a temperature of 120 °C. The results of this test were change of linear dimensions (Δl), change of geometrical volume (Δ*V*), and mass loss (Δ*m*). The values of these parameters were calculated in accordance with ISO 1923:1981 and PN-EN ISO 4590:2016-11.

Absorbability (A) and water absorption (WA) were determined and calculated in accordance with ISO 2896:2001, which were measured after immersion in distilled water for 24 h.

#### 2.5.3. Flammability Tests

Three flammability tests were performed on the new RPU/PIR foams: Bütler’s combustion test (vertical or chimney test) in accordance with ASTM D3014-04, horizontal combustion test in accordance with PN-EN ISO 3582:2002/A1:2008 and limited oxygen index (LOI) measured by using Concept Equipment apparatus (Rustington, UK), in accordance with ISO 4589.

#### 2.5.4. Composites Structure

Foam structure analysis were carried out by using a scanning electron microscope (SEM) HITACHI SU8010 (Hitachi High-Technologies Co., Tokyo, Japan). The studies were performed at an accelerating voltage of 30 kV, with working distance of 10 mm and magnification of 150×. Statistical analysis of cell sizes, wall thickness, and content of cell per area unit was carried out on the basis of obtained micrographs using ImageJ software (LOCI, Madison, WI, USA).

## 3. Results and Discussion

### 3.1. Properties of New PLA-Polyol

#### 3.1.1. Analytical Tests

The synthesis obtained a light-brown polyol without any smell. PLA-polyol was characterized by density of 1.04 g/cm^3^, viscosity of 3600 mPa·s, pH of 5, and water content of 0.331 wt %. The hydroxyl value of this compound was 326 mg KOH/g, and acid value was 18.12 mg KOH/g. The molecular weight of PLA-polyol was 723 g/mol with functionality of 4.2. The elemental composition of PLA-polyol is shown in Table 3.

The obtained analytical results were necessary to calculate the formulations of the RPU/PIR foams.

#### 3.1.2. Spectroscopy Tests

**Figure 3:**
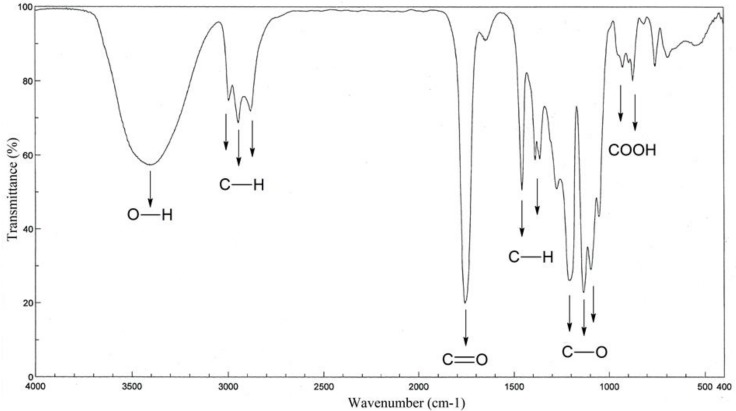
IR spectra of PLA-polyol.

Analysis of PLA-polyol in FTIR spectroscopy (Figure 3) showed that there were characteristic bonds of the structure of lactide esters. The spectrum of this polyol showed high band intensity at 3400 cm^−1^, which indicated the presence of O–H bonds in the hydroxyl groups. Bands at 2880–3000 cm^−1^ (stretching) and 1360–1460 cm^−1^ (deformational) belonged to the C–H bond of the –CH_2_– and –CH_3_ group; 1640–1760 cm^−1^ (stretching) to the C=O bond of the ester group, 1050–1270 cm^−1^ (stretching) to the C-O bond of the ester group, and 870–930 cm^−1^ to the free carboxyl group.

**Figure 4:**
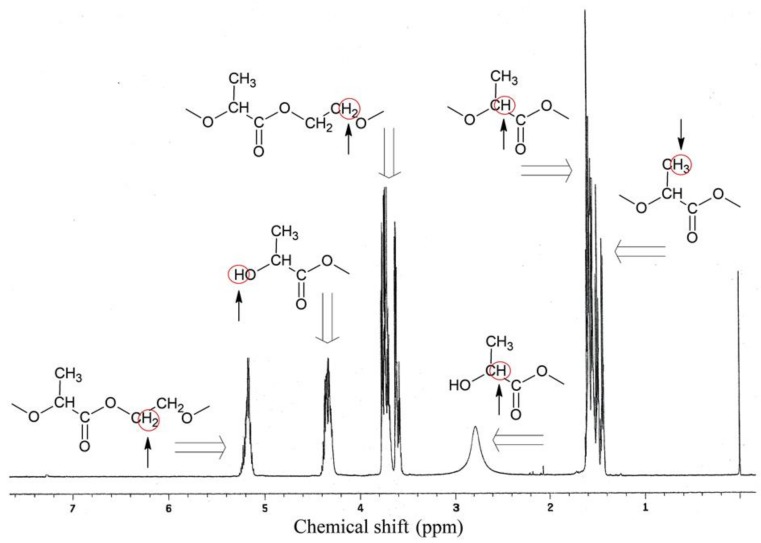
^1^H NMR spectra of PLA-polyol.

**Figure 5:**
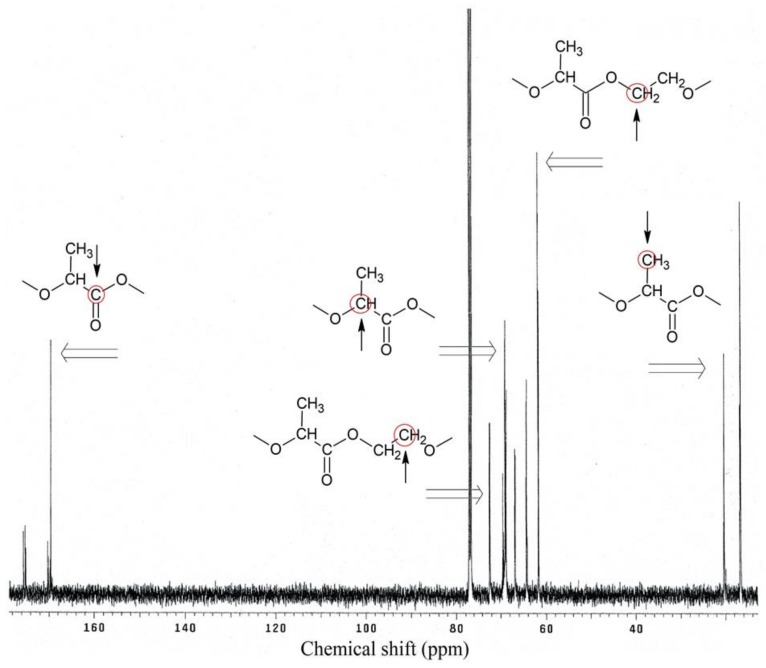
^13^C NMR spectra of PLA-polyol.

PLA-polyol analysis in nuclear magnetic resonance spectroscopy ^1^H NMR (Figure 4) and ^13^C NMR (Figure 5) confirmed the presence of characteristic groups of these compounds, resulting from its expected chemical structure (Figure 1).

^1^H NMR spectrum analysis showed characteristic chemical shifts for 1.43–1.44 ppm protons of methyl group from lactic acid monomers, 1.5–1.6 ppm protons of the α-CH_2_ group to the carbonyl group, 2.6–2.9 ppm protons of tri-substituted carbon in the α-position to the hydroxyl group (ending of polymer chain), 3.7–3.8 ppm protons of α-CH_2_ groups to the alcoxyl group (from glycol), 4.2–4.3 ppm protons of hydroxyl groups in macromolecules, and 5.17 ppm protons of α-CH_2_ groups to the ester group.

^13^C NMR spectrum analysis showed characteristic chemical shifts for 16.61–20.32 ppm carbons of methyl groups, 61.61–61.71 ppm carbons of β-CH_2_ groups to the alcoxyl group, 68.57–69.21 ppm carbons of α-CH_2_ groups to the ester group, 72.29 ppm carbons of α-CH_2_ groups to the alcoxyl group, and 169.59 ppm carbons of the carbonyl groups.

#### 3.1.3. Differential Scanning Calorimetry (DSC)

**Figure 6:**
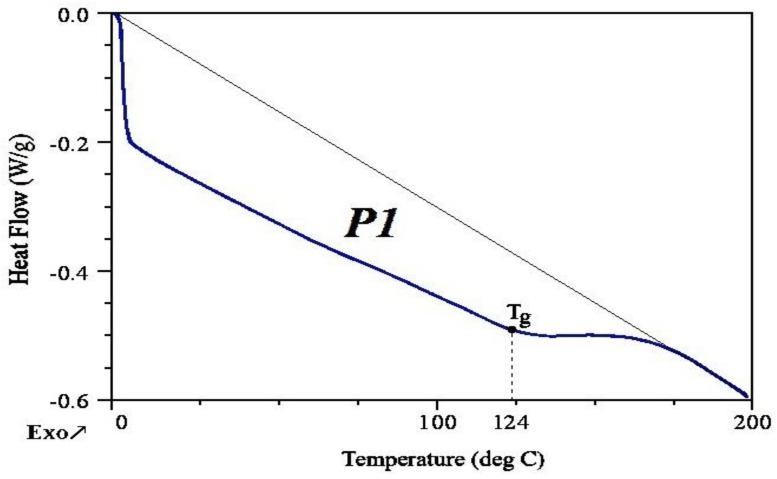
DSC curve of PLA-polyol.

The obtained polyol based on PLA waste from 3D printing was tested by differential scanning calorimetry (DSC), in accordance with industrial standards. The test was carried out in one measuring cycle in a temperature range corresponding to the real conditions of polyol processing. Analysis of the DSC curve of PLA-polyol showed the presence of only one local extreme (P1, Figure 6). It corresponded to the transition (P1) of endothermic character, associated with glass transition of PLA-polyol and, to a small extent, with the evaporation of water contained in the polyol (0.331 wt %). The glass transition temperature (*T*_g_), in which a transition from glassy state (<*T*_g_) to liquid state (>*T*_g_) takes place, was 124 °C. Heating above this range led to a gradual decomposition of the polyol [33].

### 3.2. Properties of Rigid PU/PIR Foams

#### 3.2.1. Foaming Process

Six types of foams were obtained: G3.0—foam without PLA-polyol and G3.1–G3.5—foams with increasing content of PLA-polyol (from 0.1 to 0.5 mass equivalent of OH groups) by the partial replacement of petrochemical polyol. 

The processing time taken to prepare the foams was measured (Table 4). There was no observed effect of PLA-polyol, which meant that the new polyol had no effect on the reactivity of the polyurethane mixture.

The lack of influence of polyol addition on the foaming process may be a result of similar functionalities and molecular weights of the polyols used. The functionality of PLA-polyol was 4.2 and that of Rokopol RF-551 was 4.5. While the molecular weight of recycling-based polyol was 723 g/mol, the *M*_n_ of petrochemical polyol was 650 g/mol.

#### 3.2.2. Selected Properties of New Composites

Rigid polyurethane foams are used primarily as a thermal insulation and filling material. Therefore, the vital properties of these materials are apparent density, compressive strength, absorbability, and water absorption, with which dimensional stability, thermal conductivity coefficient are also associated. An important factor determining the functional properties of polyurethane foams is their brittleness and flammability. 

The chemical structure and properties of polyol raw materials used in PU formulation significantly affect the foam structure formed and their useful properties. As a result of the addition of a new PLA-polyol, obtained on the basis of polylactide waste in polyurethane formulation, RPU/PIR foams with different apparent densities were obtained. The highest value of the apparent density was characterized by reference foam of G3.0–37.43 kg/m^3^, which was obtained on the basis of petrochemical polyol. A decrease of this parameter to 31.43 kg/m^3^ for the G3.5 foam was noted after addition of the PLA-polyol. It was found that increasing the content of the new polyol in RPU/PIR foams resulted in a decrease of density. The use of a glycolysis-based recycling technology affected the obtaining of polyol, which contained long chains with a linear structure (Figure 2). The addition of such a component into the foam structure caused a decrease in its apparent density, because there was a decrease in the degree of packing of polymer molecules and an increase in its elasticity. The usage of materials with a lower apparent density is more advantageous from an economic point of view. Similar dependence was observed by Marcovich et al., when they used vegetable-based biopolyols. Increasing the content of biopolyols to 50 weight parts in the PU formulation resulted in a decrease of apparent density from 35 kg/m^3^ to 30 kg/m^3^ [34]. Czupryński et al. used PU glycolysis products to obtain RPU/PIR foams. In this case, inverse dependence was noted. The addition of 0.3 *Eq* of PU glycolysate caused an increase of apparent density from 37 kg/m^3^ to 66 kg/m^3^ [35].

**Figure 7:**
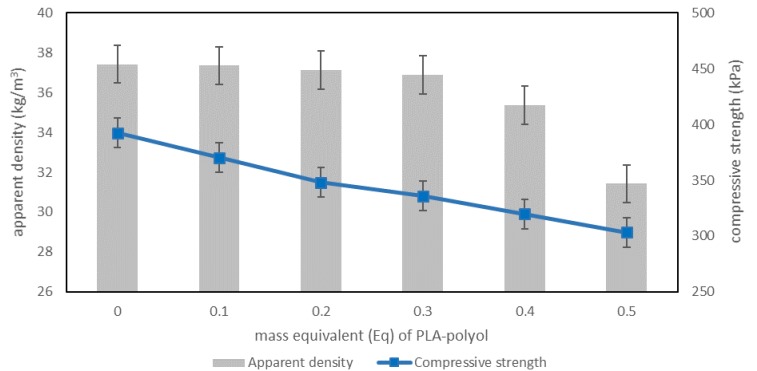
Dependence between polyol content, apparent density, and compressive strength in a direction parallel to growth of the foams.

However, the apparent density of RPU/PIR foam is in close relation to its physicomechanical as well as thermal insulation properties [36,37,38]. Increased elasticity of the polymer matrix and decreased density of the obtained RPU/PIR foams significantly affected their compressive strength (Figure 7).

A decrease in compressive strength from 392.53 kPa (G3.0) to 303.16 kPa (G3.5) was observed with an increase of PLA-polyol content in the formulation of foams. Their compressive strength values decreased with a decrease in apparent density of the new foams. However, values of this parameter were still very high, because the industry standard is at least 280 kPa for RPU/PIR foams. Moreover, taking into account the fact that the used polyol was obtained as a result of chemical recycling of waste meant that these values are indeed high. In general, polyol raw material from the glycolysis process, which was used for the production of PU materials, resulted in the creation of foams with a compressive strength well below the 300 kPa value [39,40].

The compressive strength of rigid polyurethane foam is an important parameter, because it is used as a thermal insulator that must withstand compressive loads. In addition, compressive strength affects the stability of the linear dimensions of RPU/PIR foams. The deformation of foam can often be observed when in use, particularly when it is exposed to alternating high and low temperatures. The reason for this are the pressure changes of gas filling (in this case—carbon dioxide) the foam cells, caused by temperature changes in foam ambience. The foam will not deform if its compressive strength is higher than 100 kPa, i.e., the pressure difference that can occur between atmospheric pressure and the pressure inside the foam cells (assuming total condensation of the blowing agent) [41]. Aging tests of the obtained foams was carried out within 48 h, because the most characteristic changes occur in the first 24 to 72 h of their aging. Based on the obtained results of the accelerated aging tests (Table 5), it can be concluded that there was no close relationship between dimensional stability, change of geometrical volume, mass loss, and content of PLA-polyol in the foams’ formulation. During the simulated aging of RPU/PIR foams in the dryer, the determined changes in linear dimensions and geometrical volume did not exceed 2%. Mass losses did not exceed 3.5% for all foams. The first two parameters were at the same level as when using plant-based biopolyols and PU glycolyzates for the synthesis of RPU/PIR foams. The difference was in the mass loss value. Foams based on biopolyols and PU glycolysates showed a downward trend of this parameter [28,35,40]. This is due to the presence of thermally resistant molecules from vegetable oils (in biopolyols) and complex structures containing hard segments of polyurethane (in PU glycolysates). The increase of their content caused a decrease of mass loss during the thermostatting process. In the case of PLA glycolysis products, a linear polyester was added to the polyurethane structure. This compound gradually decomposed under long exposure at higher temperature and oxidizing atmosphere (air). Therefore, an increase of mass loss was observed.

The compressive strength of rigid polyurethane foam is an important parameter, because it is used as a thermal insulator that must withstand compressive loads. In addition, compressive strength affects the stability of the linear dimensions of RPU/PIR foams. The deformation of foam can often be observed when in use, particularly when it is exposed to alternating high and low temperatures. The reason for this are the pressure changes of gas filling (in this case—carbon dioxide) the foam cells, caused by temperature changes in foam ambience. The foam will not deform if its compressive strength is higher than 100 kPa, i.e., the pressure difference that can occur between atmospheric pressure and the pressure inside the foam cells (assuming total condensation of the blowing agent) [41]. Aging tests of the obtained foams was carried out within 48 h, because the most characteristic changes occur in the first 24 to 72 h of their aging. Based on the obtained results of the accelerated aging tests (Table 5), it can be concluded that there was no close relationship between dimensional stability, change of geometrical volume, mass loss, and content of PLA-polyol in the foams’ formulation. During the simulated aging of RPU/PIR foams in the dryer, the determined changes in linear dimensions and geometrical volume did not exceed 2%. Mass losses did not exceed 3.5% for all foams. The first two parameters were at the same level as when using plant-based biopolyols and PU glycolyzates for the synthesis of RPU/PIR foams. The difference was in the mass loss value. Foams based on biopolyols and PU glycolysates showed a downward trend of this parameter [28,35,40]. This is due to the presence of thermally resistant molecules from vegetable oils (in biopolyols) and complex structures containing hard segments of polyurethane (in PU glycolysates). The increase of their content caused a decrease of mass loss during the thermostatting process. In the case of PLA glycolysis products, a linear polyester was added to the polyurethane structure. This compound gradually decomposed under long exposure at higher temperature and oxidizing atmosphere (air). Therefore, an increase of mass loss was observed.

The new polyols used to obtain rigid polyurethane-polyisocyanurate foams had a significant impact on their brittleness (Table 6). The number of elastic segments in the polyurethane structure increased with the increase of PLA-polyol content in the formulation, which in effect caused a decrease in its stiffness. It is known that polyester-based polyols have more elastic segments than polyether-based polyols. This is due to their chemical structure. Therefore, the addition of linear polyesterol in place of petrochemical polyetherol promotes a decrease in the stiffness of the obtained materials. A consequence of this was a significant decrease of brittleness of the RPU/PIR foams modified by recycling-based polyol. Values of brittleness changed on increasing the content of new polyols from 32.3% for the G3.0 (0.0 *Eq* of PLA-polyol) foam to 0.6% for the G3.5 (0.5 *Eq*) foam. The same trend was noted by using biopolyols based on plant raw materials in similar RPU/PIR systems. The addition of 0.5 *Eq* of biopolyol resulted in a decrease of brittleness from 40% to 3.5% [42]. However, the use of polyols based on PU glycolysis had an opposite effect—the increase of brittleness with increasing content. The decrease of this parameter is beneficial from an application point of view [43].

The use of a PLA polyol in the formulation of foams contributed to changes in absorbability and water absorption values (Table 6). It was noted that modifying the formulation by the addition of a new polyol led to a slight decrease in absorbability—from 19.42% for the reference foam G3.0 to 16.02% for the G3.5 foam. On the other hand, the significant effect of PLA-polyol addition on water absorption parameter was observed. Foams with water absorption lower than 60% to 80% in relation to the reference foam were obtained following the modification. The low water absorption capacity of RPU/PIR foams is an advantageous feature, because it allows the use of these foams as insulating material in places with high humidity, without worrying about the decrease of insulation properties [44]. A similar decrease of absorbability (from 23% to 1.5%) and water absorption (from 3.5% to 1%) was observed by using raw materials based on vegetable oils. In this case, it resulted from the addition of long chains of hydrophobic fatty acid residues to the polyurethane molecule [27,28]. However, the use of PU glycolysates increased these parameters. The reason for this was the complex structure of the recycled polyol molecule that promoted the formation of open cell foams [40]. The increase of absorbability and water absorption is an undesirable phenomenon, because it causes a decrease in the thermal insulation properties of PU foams [45].

The most important property of rigid PU foams for thermal insulation is the value of the thermal conductivity coefficient (λ). The value of this parameter depends to a large extent on the cell structure of the finished product. The thermal conductivity coefficient of the obtained foams (both reference and modified) with PLA-polyol was approx. 34.1 mW/(m·K). Therefore, the usage of a new polyol did not affect the thermal conductivity of RPU/PIR foams. The obtained value is within the industrial standard range for this type of materials, which is 30–40 mW/(m·K) [43]. Typically, the replacement of a petrochemical polyol raw material by a vegetable oil-based or a PU glycolysis-based polyol results in RPU foams with higher thermal conductivity coefficient (35–40 mW/(m·K)) [34,35,40]. It is also worth noting that the value of this parameter was higher than the λ value in commercially used materials. This is due to the fact that carbon dioxide (obtained in situ from reaction of excess NCO groups of polyisocyanate and distilled water) was used as a blowing agent. This compound is safer for producers than a pentane mixture and is more environmentally friendly than freons (lower GWP and ODP potential) [46].

#### 3.2.3. Flammability Tests

Polyurethane materials, which are used in civil engineering, are required to have the highest flame-retardancy. Rigid polyurethane foams are flammable materials due to their organic polymer matrix and cellular structure. Typically, flame retardants are used in PU foams to drastically reduce their flammability [47,48,49]. The flammability test of the obtained RPU/PIR foams was carried out by three methods: Limited oxygen index method, residue after combustion in the vertical test, and burning in a horizontal position. These methods made it possible to compare the flammability of these materials before and after modification. The LOI value of the reference foam was 24.1%, while for the foam with the highest content of PLA-polyol, it was 24.3%. The slight increase in oxygen index could be caused by the increased amount of the flame retardant compound in the foams’ formulation (Table 2). A similar correlation could be seen in the case of combustion residue values—it increased from 89.61% for the G3.0 foam to 98.04% for the G3.5 foam. The results of the flammability tests (Table 7) helped identify the foams based on PLA-polyol as flame retardant and self-extinguishing materials.

#### 3.2.4. Composite Structure

**Figure 8:**
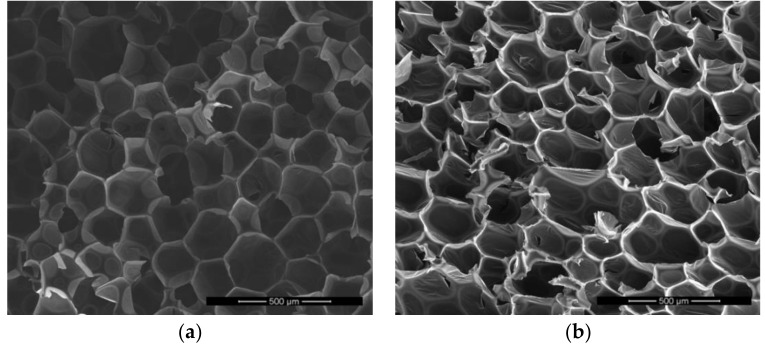
SEM micrographs of: (**a**) reference foam—G3.0, (**b**) foam modified by 0.5 Eq of PLA-polyol—G3.5.

The excellent thermal insulation properties of rigid PU foams result from the low thermal conductivity of the used blowing agent and suitable cellular structure. The thermal insulation properties of this materials can be improved by changing the shape and dimensions of its cells. In this research, carbon dioxide was used as a blowing agent. As a result, this gas was found inside the cells of the obtained foam. It should be remembered that the carbon dioxide molecule is small; therefore, the cell walls of polyurethane do not constitute a barrier. It can diffuse outside the foam and air can take its place. This mechanism causes a decrease in thermal insulation properties of polyurethane. This can be prevented by covering the polyurethane elements with protective coatings [43]. Thirumal et al. found that lower foam density caused smaller wall thickness. In addition, walls with a smaller thickness contributed to heat transfer to a small extent through the polyurethane matrix, thus decreasing the value of the thermal conductivity coefficient of the polyurethane foam [50]. The analysis and correlation of cell structure parameters of the foams with functional properties are an important part of the conducted research, because the properties of RPU/PIR foams are strongly dependent on the cellular structure. Sections of the obtained PU materials were analyzed by using scanning electron microscopy (SEM) to determine differences in the size and shape of the cells. Examples of the SEM micrographs are shown in Figure 8.

The micrographs of the obtained foams show that the cellular structure of the modified and unmodified foams was homogeneous. However, PLA-polyol contributed to the production of polyurethane-polyisocyanurate foam with cells that are slightly larger and elongated in the direction of foam growth, than the cells of the reference foam. The results of this analysis and comparison of G3.0 and G3.5 micrographs i.e., average cell size, average cell wall thickness, shape of cells and average amount of cell per area unit, are shown in Table 8. 

Cell elongation was caused by slightly longer growth time (free rise time in Table 4) and gelling time (tack free time in Table 4) of the modified foams. This structure confirmed the lower apparent density and compressive strength of foams containing PLA-polyol. At the same time, the thicker walls of these foams (resulting from the use of a linear polyol) contributed to a significant reduction in their brittleness.

## 4. Conclusions

The conditions for the chemical recycling of polylactide waste from 3D printing technology were developed as part of the research. An oligomeric polyhydric alcohol (PLA-polyol) was obtained as a result of glycolysis reaction of PLA waste by using diethylene glycol. Afterwards, this polyol raw material was used to prepare new formulations of RPU/PIR foams. The characteristic properties of polyols, such as hydroxyl number, viscosity, density, water content etc., were determined. The obtained polyol raw material was characterized by a hydroxyl number of 326 mg KOH/g, acid number of 18.12 mg KOH/g and water content of 0.331 wt %. Its functionality and molecular weight were 4.2 and 723 g/mol, respectively. The obtained parameters were similar to the properties of used petrochemical polyol (Rokopol RF-551). The chemical structure of the new compound was confirmed by the following analyzes: ^1^H NMR, ^13^C NMR and FTIR. The obtained glycolysis product was used as a polyol raw material for the synthesis of RPU/PIR foams. The foams were obtained by a one-step method, from a two-component system in mass equivalent ratio of NCO:OH groups equal to 3.7:1. The use of PLA-polyol in a mixture with petrochemical polyol allowed to obtain materials, with similar properties as commercially available foams. The increase in content of the new polyol (to 0.5 Eq) in polyurethane foams caused a slight elongation of technological time. The modification of polyurethane materials by PLA-based polyol resulted in a significant decrease of density (from 37.43 for G3.0 to 31.43 kg/m^3^ for G3.5) and compressive strength (from 392.53 kPa for G3.0 to 303.16 kPa for G3.5).However, a decrease in absorbability (from 19.42 to 16.02 wt %) and water absorption (from 5.98 to 1.17 wt %) was a positive effect. The addition of non-branched chain polyol significantly reduced brittleness from 32.13% for the reference foam to 0.60% for the foam with the highest content of recycling-based polyol. It also had a positive effect on aging resistance. The PLA-polyol used in the formulation did not affect the flammability of the foams. It was found that the developed method allowed fast, cheap and ecological management of polylactide waste with the possibility of its reuse in another form, for example, as a thermal-insulating polyurethane material.

## 5. Patents

The synthesis of PLA-polyol based on 3D printing waste and synthesis of RPU/PIR foams base on this polyol were carried out on the basis of the patented methods—Polish Patent Application numbers: P.424629 and P.424630.

## Figures and Tables

**Table 1:** Amount of the reactants in the glycolysis process.

Ground PLA (g)	98% Diethylene Glycol (g)	Zinc Stearate (g)
1000.00	500.00	2.00

**Table 2:** Formulation of RPU/PIR foams with PLA-polyol.

Foam Symbol	Rokopol RF-551, *(Eq)* (g)	PLA Polyol *(Eq)* (g)	Tegostab 8460 (g)	33% DABCO (g)	33% Potassium Acetate (g)	Antiblaze TCMP (g)	Distilled Water *(Eq)* (g)	Purocyn B *(Eq)* (g)
**G3.0**	*1.0* 66.80	*0.0* 0.00	5.40	3.15	7.95	54.00	*0.7* 3.15	*3.7* 250.60
**G3.1**	*0.9* 60.12	*0.1* 8.60	5.42	3.19	7.96	54.22	*0.7* 3.19	*3.7* 250.60
**G3.2**	*0.8* 53.44	*0.2* 17.21	5.45	3.21	8.03	54.54	*0.7* 3.21	*3.7* 250.60
**G3.3**	*0.7* 46.76	*0.3* 25.81	5.49	3.23	8.08	54.87	*0.7* 3.23	*3.7* 250.60
**G3.4**	*0.6* 40.08	*0.4* 34.42	5.52	3.25	8.13	55.20	*0.7* 3.25	*3.7* 250.60
**G3.5**	*0.5* 33.4	*0.5* 43.02	5.55	3.27	8.18	55.53	*0.7* 3.27	*3.7* 250.60

**Table 3:** Results of elemental analysis of PLA-polyol.

Element	Carbon (%)	Hydrogen (%)	Oxygen (%)
PLA-polyol	48.31 ± 0.18	8.72 ± 0.13	42.97 ± 0.21

**Table 4:** Processing time of RPU/PIR foams with PLA-polyol.

Foam Symbol	Cream Time (s)	String Gel Time (s)	Tack Free Time (s)	Free Rise Time (s)
**G3.0**	10	20	23	40
**G3.1**	12	22	25	42
**G3.2**	12	22	25	42
**G3.3**	12	22	25	42
**G3.4**	12	22	25	42
**G3.5**	12	26	25	42

**Table 5:** The aging tests results.

Parameter	G3.0	G3.1	G3.2	G3.3	G3.4	G3.5
**Change of linear dimensions (%)**	1.73 ± 0.18	1.29 ± 0.04	1.22 ± 0.18	1.00 ± 0.11	0.80 ± 0.05	0.40 ± 0.04
**Change of geometric volume (%)**	0.47 ± 0.09	1.46 ± 0.17	1.91 ± 0.16	1.67 ± 0.36	1.73 ± 0.14	1.39 ± 0.28
**Mass loss (%)**	1.00 ± 0.05	0.88 ± 0.07	1.68 ± 0.23	2.28 ± 0.38	2.33 ± 0.34	3.24 ± 0.39

**Table 6:** Performance properties of PLA-RPU/PIR composites.

Parameter	G3.0	G3.1	G3.2	G3.3	G3.4	G3.5
**Brittleness (%)**	32.13 ± 2.14	16.34 ± 1.03	9.59 ± 0.81	2.95 ± 0.19	1.53 ± 0.18	0.60 ± 0.03
**Absorbability (%)**	19.42 ± 0.26	17.22 ± 0.58	16.75 ± 0.81	16.73 ± 0.76	16.43 ± 0.92	16.02 ± 0.76
**Water absorption (%)**	5.98 ± 0.61	2.25 ± 0.21	1.60 ± 0.17	1.55 ± 0.10	1.42 ± 0.16	1.17 ± 0.11

**Table 7:** Flammability tests results.

Parameter	G3.0	G3.1	G3.2	G3.3	G3.4	G3.5
**Combustion residue (%)**	89.61 ± 0.48	91.96 ± 0.21	94.97 ± 0.16	95.36 ± 0.51	97.96 ± 0.63	98.04 ± 0.39
**LOI (% vol. of O_2_)**	24.0 ± 0.1	24.1 ± 0.1	24.1 ± 0.1	24.2 ± 0.2	24.3 ± 0.1	24.3 ± 0.2
**Classification based on** **PN-EN ISO 3582:2002**	self-extinguishing					

**Table 8:** Results of SEM micrographs analysis.

Foam Symbol	Cell Size (μm)	Thickness of Cell Wall (μm)	Content of Cell per Area Unit (cell/mm^2^)
G3.0	213 ± 15	17 ± 2	13 ± 1
G3.5	222 ± 18	18 ± 2	12 ± 2
